# *Naegleria fowleri* Extracellular Vesicles Induce Proinflammatory Immune Responses in BV-2 Microglial Cells

**DOI:** 10.3390/ijms241713623

**Published:** 2023-09-03

**Authors:** Hương Giang Lê, Jung-Mi Kang, Tuấn Cường Võ, Won Gi Yoo, Byoung-Kuk Na

**Affiliations:** 1 Department of Parasitology and Tropical Medicine, Institute of Medical Science, College of Medicine, Gyeongsang National University, Jinju 52727, Republic of Koreajmkang@gnu.ac.kr (J.-M.K.); vtcuong241@gmail.com (T.C.V.); wgyoo@gnu.ac.kr (W.G.Y.); 2 Department of Convergence Medical Science, Gyeongsang National University, Jinju 52727, Republic of Korea

**Keywords:** *Naegleria fowleri*, extracellular vesicles, microglial cells, proinflammatory response

## Abstract

Extracellular vesicles (EVs) of protozoan parasites have diverse biological functions that are essential for parasite survival and host–parasite interactions. In this study, we characterized the functional properties of EVs from *Naegleria fowleri*, a pathogenic amoeba that causes a fatal brain infection called primary amoebic meningoencephalitis (PAM). *N. fowleri* EVs (NfEVs) have been shown to be internalized by host cells such as C6 glial cells and BV-2 microglial cells without causing direct cell death, indicating their potential roles in modulating host cell functions. NfEVs induced increased expression of proinflammatory cytokines and chemokines such as TNF-α, IL-1α, IL-1β, IL-6, IL-17, IFN-γ, MIP-1α, and MIP-2 in BV-2 microglial cells; these increases were initiated via MyD88-dependent TLR-2/TLR-4. The production levels of proinflammatory cytokines and chemokines in NfEVs-stimulated BV-2 microglial cells were effectively downregulated by inhibitors of MAPK, NF-κB, or JAK-STAT. Phosphorylation levels of JNK, p38, ERK, p65, JAK-1, and STAT3 were increased in NfEVs-stimulated BV-2 microglial cells but were effectively suppressed by each corresponding inhibitor. These results suggest that NfEVs could induce proinflammatory immune responses in BV-2 microglial cells via the NF-κB-dependent MAPK and JAK-STAT signaling pathways. Taken together, these findings suggest that NfEVs are pathogenic factors involved in the contact-independent pathogenic mechanisms of *N. fowleri* by inducing proinflammatory immune responses in BV-2 microglial cells, further contributing to deleterious inflammation in infected foci by activating subsequent inflammation cascades in other brain cells.

## 1. Introduction

Extracellular vesicles (EVs) are lipid membrane-enclosed vesicular structures released from living cells that play crucial roles in intercellular communication between distant cells [1,2]. EVs are typically classified into three types, microvesicles, exosomes, and apoptotic bodies, depending on their size, composition, release pathway, biogenesis, and biological function [1,2]. EVs contain various components, such as lipids, proteins, nucleic acids, and membrane receptors originating from cells. EVs enable the transfer of these molecules to other cells or target cells via internalization, resulting in physiological changes in recipient cells [2,3]. Studies on protozoan parasites have reported critical functions of parasite EVs in virulence factor delivery, interactions with host cells, and host immune modulation [4,5,6]. EVs from *Leishmania* spp. are involved in the pathogenicity of the parasites by facilitating their invasion into host cells, delivering virulence factors and drug-resistance genes, and modulating host–pathogen communication [7,8,9,10]. Exosomes released from *Trichomonas vaginalis* can enhance attachment of the parasite to host cells and modulate host immune responses by downregulating IL-8 secretion [11]. *Trypanosoma-brucei*-derived EVs transfer their cargo to host red blood cells (RBC) and cause physiological alterations in the RBC membrane, eventually inducing cell clearance [12]. EVs from *Acanthamoeba castellanii* are known to contribute to the pathogenesis of amoebae by inducing a change in the cell adhesion ability of the parasite and subsequent host cell death [13,14].

*Naegleria fowleri* is an amoeba that causes a fatal brain infection called primary amoebic meningoencephalitis (PAM) in humans. Although global PAM cases are rare, mortality rate of this disease is extremely high, reaching up to 97% [15]. Once *N. fowleri* trophozoites infect humans via the nasal route, they penetrate host nasal nerve cells and invade the central nervous system (CNS) via the olfactory nerves, thereby resulting in severe inflammatory and hemorrhagic damage in the brain [16]. Direct phagocytic attacks by actively proliferating trophozoites and indirect cytolytic effects or inflammatory attacks by molecules secreted from the amoeba have been proposed as the major pathogenic mechanisms that induce host cell damage and inflammation in the brain [16,17,18,19,20]. However, the molecular pathogenic mechanisms induced by amoebae are poorly understood and, therefore, need to be investigated further. In this study, we investigated the EVs of *N. fowleri* (NfEVs) as pathogenic factors that mediate proinflammatory immune responses in microglial cells.

## 2. Results

### 2.1. Biophysical Characteristics of NfEVs

**Figure 1:**
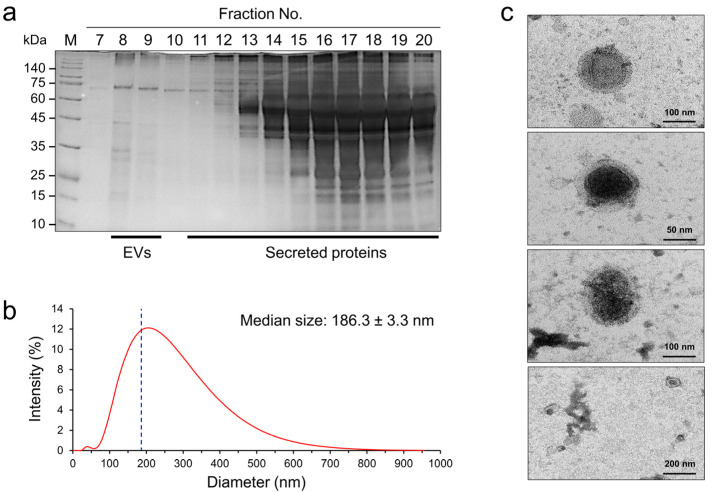
Purification and biophysical properties of NfEVs: (**a**) Purification of NfEVs. NfEVs were purified from culture media of actively proliferating *N. fowleri* trophozoites by using qEV column. Each fraction (500 μL) was collected and analyzed using SDS–PAGE and performed silver staining. Fractions 8 and 9, which contained enriched NfEVs, were pooled and used for further study. (**b**) Dynamic light scattering (DLS) analysis. DLS plots represented the wide range of size variations of NfEVs with a median size of 186.3 ± 3.3 nm. (**c**) Transmission electron microscopic analysis. NfEVs had round shapes with typical bilayered membrane structures.

SDS–PAGE analysis of the fractions eluted from the qEV column showed that fractions 8 and 9 contained enriched NfEVs, with the highest concentration in fraction 8 (Figure 1a). Fractions 11–20 contained the secreted proteins. DLS analysis of NfEVs revealed that the sizes of the NfEVs ranged from 22.4 to 955 nm with a median of 186.3 ± 3.3 nm (Figure 1b). The highest intensity of NfEVs size was 220 nm, accounting for approximately 12%. TEM analysis of NfEVs revealed that the vesicles had a rounded shape with clear bilayered membrane structures (Figure 1c).

### 2.2. Internalization of NfEVs into Mammalian Cells

**Figure 2:**
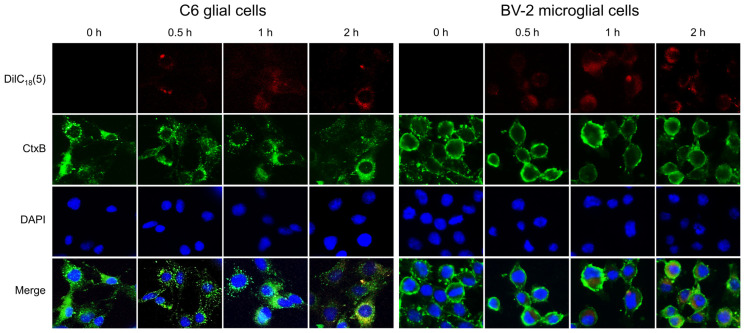
Internalization of NfEVs into mammalian cells. NfEVs (100 µg/mL) labeled with DilC_18_(5) (red) were incubated with C6 glial cells or BV-2 microglial cells for different time points. After 3 washes with PBS, the cells were stained with CtxB-Alexa (green) and DAPI (blue). NfEVs were identified in the cytoplasm of the cells, indicating NfEVs were internalized into the mammalian cells. The images were observed using an EVOS M5000 Imaging System at a magnification of ×40. Images are representative of the cell populations observed in three individual experiments.

To investigate whether NfEVs could be internalized by mammalian cells, DilC_18_(5)-labeled NfEVs were incubated with C6 glial cells and BV-2 microglial cells. After 30 min of incubation, the attachment of NfEVs to recipient cells was detected (Figure 2). The red fluorescence of DilC_18_(5)-labeled NfEVs was partially colocalized with the green fluorescence of CtxB in the cytoplasm of both C6 glial cells and BV-2 microglial cells after 1 h of incubation. Clear internalization of NfEVs into cells was observed 2 h post-incubation, particularly in BV-2 microglial cells (Figure 2). Meanwhile, no signal indicating non-specific attachment and internalization of DilC_18_(5) was detected in both cells treated with only DilC_18_(5) (Supplement File S1: Figure S1). These findings suggest that NfEVs are fused with cells or engulfed by cells and that the contents are consequently dispersed into the cells. A cell viability assay was performed to evaluate the effects of NfEVs on the viability of C6 glial, CHO-K1, and BV-2 microglial cells. The morphology of these cells was not changed by NfEVs even after treatment with high concentrations of NfEVs (Supplement File S2: Figure S2a). Moreover, NfEVs did not have any cytopathic effects on cells (Supplement File S2: Figure S2b).

### 2.3. NfEVs Stimulate the Proinflammatory Immune Responses in BV-2 Microglial Cells

**Figure 3:**
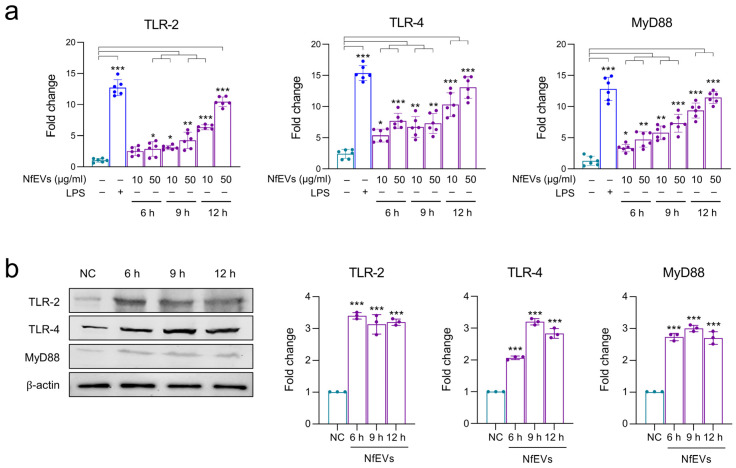
NfEVs stimulate BV-2 microglial cells via MyD88-dependent TLR-2/TLR-4. (**a**) mRNA expression. BV-2 microglial cells were treated with different concentrations (10 µg/mL or 50 µg/mL) of NfEVs for different time points (6 h, 9 h, or 12 h). Expression levels of TLR-2, TLR-4, and downstream adaptor MyD88 were analyzed using RT-qPCR. Bar graphs show the quantitative expression pattern of each gene analyzed as a fold induction of each gene relative to *nfgapdh* in three independent experiments. A one-way ANOVA with Dunnett’s post hoc test was performed as multiple comparisons with the negative control without treatment with either LPS or NfEVs. *** *p* < 0.0001, ** *p* < 0.001, and * *p* < 0.01. (**b**) Protein expression. Expressions of TLR-2, TLR-4 and MyD88 were analyzed in NfEVs-treated BV-2 microglial cells using immunoblots. NC: negative control without treatment of NfEVs. β-actin was used as an internal control. Fold change (mean ± SD) means relative density change compared to negative control without treatment with NfEVs. *** *p* < 0.0001.

**Figure 4:**
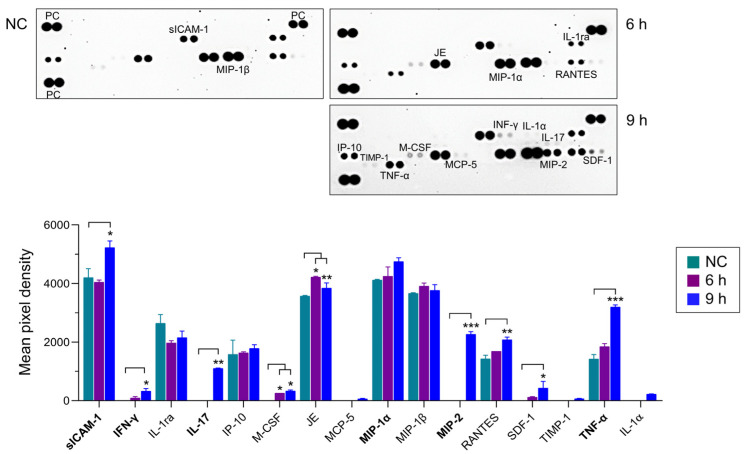
Cytokine profiles of BV-2 microglial cells stimulated by NfEVs. BV-2 microglial cells were stimulated with NfEVs (50 µg/mL) and the cell supernatants were analyzed using the Proteome Profiler Mouse Cytokine Array kit. The culture supernatant from the cells without treatment of NfEVs was used as a negative control. PC is a positive reference to verify the reaction. Each cytokine or chemokine represents double dots. The density of dots was calculated by using ImageJ software. Values are presented as mean ± SD of three independent experiments. A one-way ANOVA with Dunnett’s post hoc test was performed as multiple comparisons with the control treated with NfEVs. *** *p* < 0.0001, ** *p* < 0.001, and * *p* < 0.01.

**Figure 5:**
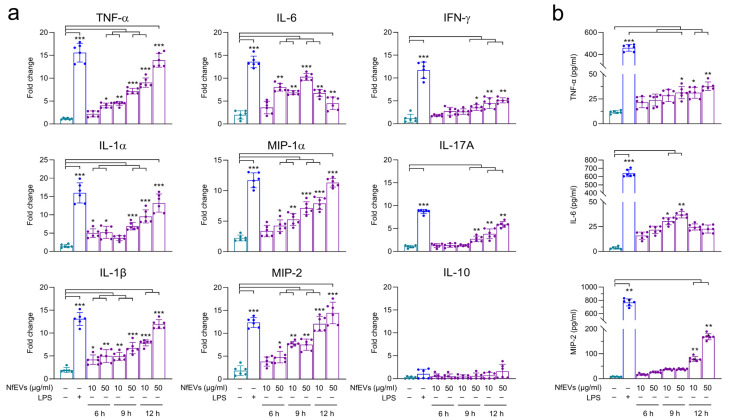
NfEVs induce productions of proinflammatory cytokines and chemokines in BV-2 microglial cells. (**a**) mRNA expression. BV-2 microglial cells were treated with different concentrations of NfEVs (10 µg/mL or 50 µg/mL) for different time points (6 h, 9 h, or 12 h). RT-qPCR was performed to analyze the expression pattern of each cytokine and chemokine. Bar graphs indicate the quantitative expression profile of each gene as a fold induction of each gene relative to *nfgapdh* in three independent experiments. (**b**) Quantitative ELISA. BV-2 microglial cells were treated with different concentrations of NfEVs for different time points. At indicated time points, the supernatant was collected and protein levels of TNF-α, IL-6, and MIP-2 were analyzed using ELISA. Values were presented as mean ± SD of three independent experiments. A one-way ANOVA with Dunnett’s post hoc test was performed as multiple comparisons with the negative control without treatment with either LPS or NfEVs. *** *p* < 0.0001, ** *p* < 0.001, and * *p* < 0.01.

Next, the immune responses in BV-2 microglial cells treated with NfEVs were analyzed. The gene expression of TLR-2, TLR-4, and adaptor MyD88 was remarkably increased in NfEVs-treated BV-2 microglial cells in a time-dependent manner (Figure 3a). The protein expression of TLRs and MyD88 also increased until 9 h post-treatment with NfEVs (Figure 3b), suggesting that NfEVs initially stimulated the immune responses of the cells via MyD88-dependent TLR-2/TLR-4. Cytokine array analysis revealed that NfEVs induce the production of diverse cytokines and chemokines in BV-2 microglial cells. The levels of sICAM-1, IL-17, MIP-2, RANTES, and TNF-α were increased by treatment with NfEVs compared to those in the negative control without treatment with NfEVs. The expressions of other cytokines and chemokines, including IFN-γ, IL-1α, IP-10, M-CSF, MIP-1α, SDF-1, and TIMP-1, were also slightly increased by NfEVs (Figure 4). However, the increase in cytokine production was not high at short treatment less than 3 h, so we selected 6 h treatment of NfEVs to BV-2 microglial cells for further analyses. The enhanced expression levels of cytokines (TNF-α, IL-1α, IL-1β, IL-6, IL-10, IL-17A, and IFN-γ) and chemokines (MIP-1α and MIP-2) at the mRNA level were further confirmed using RT-qPCR. The expression levels of these genes, except IL-10, increased significantly in both time-dependent and dose-dependent manners in NfEVs-treated BV-2 microglial cells (Figure 5a). Consistent with the mRNA expression patterns, the protein levels of TNF-α, IL-6, and MIP-2 also increased in NfEVs-treated cells (Figure 5b).

### 2.4. NfEVs Induce the Proinflammatory Immune Responses in BV-2 Microglial Cells via MAPK Signaling Pathway

**Figure 6:**
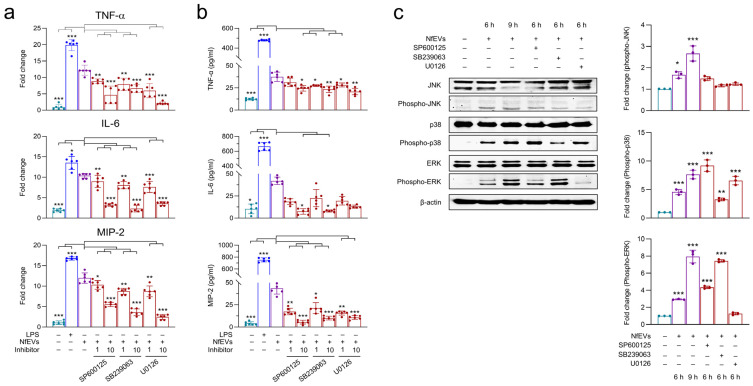
MAPK signaling pathways are involved in proinflammatory immune response of BV-2 microglial cells stimulated by NfEVs. (**a**) mRNA expression. BV-2 microglial cells were pre-treated with different concentrations (1 µM or 10 µM) of JNK inhibitor (SP600125), p38 inhibitor (SB239063), or ERK inhibitor (U0126) for 3 h. NfEVs (50 µg/mL) were then applied to the cells for 6 h. The mRNA expressions of TNF-α, IL-6, and MIP-2 were analyzed using RT-qPCR. Bar graphs indicate the quantitative expression profile of each gene as a fold induction of each gene relative to *nfgapdh* in three independent experiments. (**b**) Quantitative ELISA. Productions of TNF-α, IL-6, and MIP-2 were measured using ELISA. Values are presented as mean ± SD of three independent experiments. A one-way ANOVA with Dunnett’s post hoc test was performed as multiple comparisons with the control treated with NfEVs. (**c**) Phosphorylation levels of JNK, p38, and ERK in NfEVs-stimulated BV-2 microglial cells. To analyze phosphorylation levels of MAPKs, BV-2 microglial cells were pre-treated with JNK, p38, or ERK inhibitor (10 µM), respectively, and treated with NfEVs (50 µg/mL) for 6 h. Total proteins were extracted from the cells, and phosphorylation levels of JNK, p38, and ERK were analyzed via immunoblots using a specific antibody for each protein. The total JNK, p38, ERK, and β-actin were used as internal controls. Fold change (mean ± SD) means relative density change compared to negative control without treatment with NfEVs and inhibitor. *** *p* < 0.0001, ** *p* < 0.001, and * *p* < 0.01.

To investigate whether the MAPK signaling pathway is involved in proinflammatory immune responses in BV-2 microglial cells stimulated with NfEVs, the cells were pre-treated with different concentrations of each inhibitor and further incubated with NfEVs. The mRNA expression of cytokines was found to be notably suppressed by pre-treatment with 10 μM of JNK, p38, or ERK inhibitor (Figure 6a). The protein levels of IL-6 and MIP-2 were also reduced by each inhibitor in a dose-dependent manner, but the reduction of TNF-α was not significant (Figure 6b). Immunoblot analysis revealed that NfEVs enhanced the phosphorylation of JNK, p38, and ERK. The phosphorylation status of JNK, p38, and ERK was reduced by pre-treatment with the corresponding inhibitors (Figure 6c).

### 2.5. NfEVs-Induced Proinflammatory Immune Responses in BV-2 Microglial Cells Are Regulated by NF-κB Signaling Pathway

**Figure 7:**
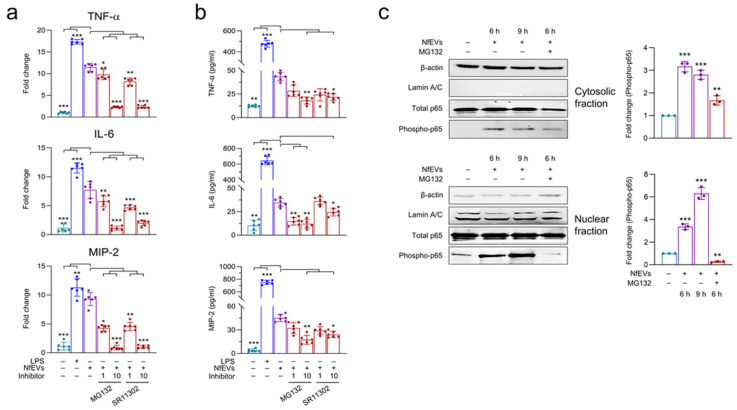
Effects of NF-κB and AP-1 inhibitors on proinflammatory immune response of BV-2 microglial cells stimulated by NfEVs. (**a**) mRNA expression. BV-2 microglial cells were pre-treated with different concentrations (1 µM or 10 µM) of NF-κB inhibitor (MG132) or AP-1 inhibitor (SR11302) for 3 h, and treated with NfEVs (50 µg/mL) for 6 h. mRNA expression levels of TNF-α, IL-6, and MIP-2 were analyzed using RT-qPCR. Bar graphs indicate the quantitative expression profile of each gene as the fold induction of each gene relative to *nfgapdh* in three independent experiments. (**b**) Quantitative ELISA. Productions of TNF-α, IL-6, and MIP-2 were measured using ELISA. Values are presented as mean ± SD of three independent experiments. A one-way ANOVA with Dunnett’s post hoc test was performed as multiple comparisons with the control treated with NfEVs. (**c**) Phosphorylation and nuclear translocation of p65 in NfEVs-stimulated BV-2 microglial cells. To analyze the phosphorylation level and translocation of p65, BV-2 microglial cells were treated with NfEVs (50 µg/mL) with or without a pre-treatment of MG132. Cytoplasmic proteins and nuclear proteins were extracted from the cells separately. Phosphorylation of p65 was analyzed via immunoblots using a specific antibody for each protein. The β-actin and lamin A/C were used as internal controls. Fold change (mean ± SD) means relative density change compared to negative control without treatment with NfEVs and inhibitor. *** *p* < 0.0001, ** *p* < 0.001, and * *p* < 0.01.

The mRNA expression of TNF-α, IL-6, and MIP-2 was significantly decreased by pre-treatment with inhibitors, NF-κB (MG132) and AP-1 (SR11302) (Figure 7a). Pre-treatment with MG132 resulted in a significant decrease in the protein levels of cytokines and chemokines (Figure 7b). SR11302 also caused a reduction in protein levels; however, this trend was not statistically significant (Figure 7c). NfEVs enhanced the phosphorylation of p65 both in the cytosol and nucleus. Phosphorylation of p65 was suppressed by treatment with MG132, suggesting that the NF-κB signaling pathway is the major pathway that induces proinflammatory immune responses in BV-2 microglial cells upon treatment with NfEVs (Figure 7c).

### 2.6. JAK-STAT Signaling Pathway Is Also Involved in NfEVs-Induced Proinflammatory Immune Responses in BV-2 Microglial Cells

**Figure 8:**
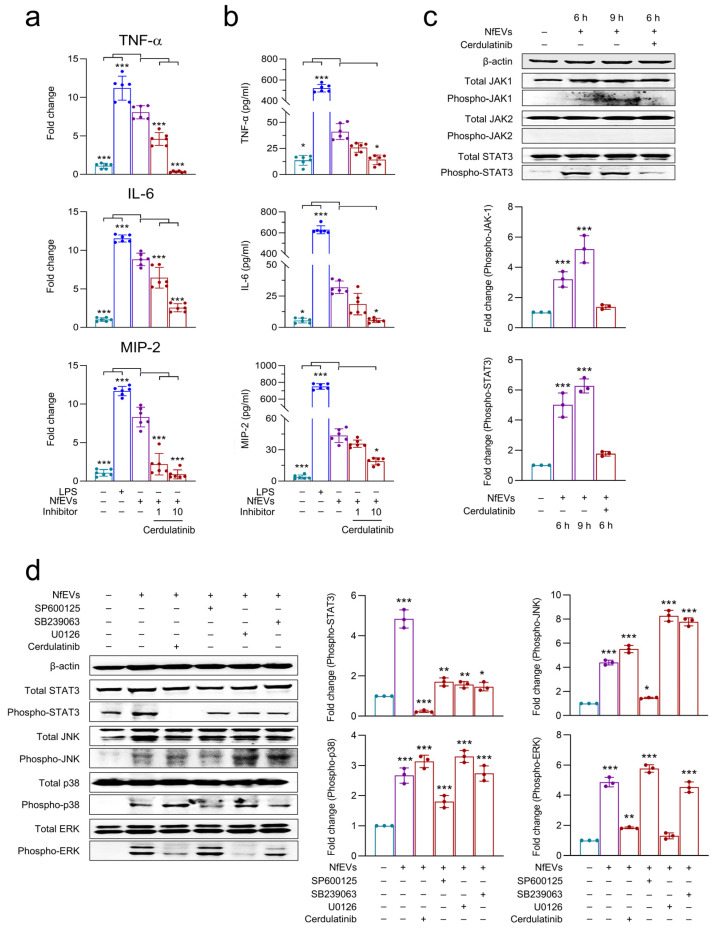
JAK-STAT signaling pathway regulates proinflammatory immune response of BV-2 microglial cells stimulated by NfEVs. (**a**) mRNA expression. BV-2 microglial cells were pre-treated with different concentrations (1 µM or 10 µM) of a JAK-STAT inhibitor (cerdulatinib) for 3 h, followed by treatment with NfEVs (50 µg/mL) for 6 h. The mRNA expression levels of TNF-α, IL-6, and MIP-2 were analyzed using RT-qPCR. Bar graphs indicate the quantitative expression profile of each gene as the fold induction of each gene relative to *nfgapdh* in three independent experiments. (**b**) Quantitative ELISA. The productions of TNF-α, IL-6, and MIP-2 were measured using ELISA. Values are presented as mean ± SD of three independent experiments. A one-way ANOVA with Dunnett’s post hoc test was performed as a multiple comparisons test with the control treated with NfEVs. (**c**) Phosphorylation levels of JAK1, JAK2, and STAT3 in NfEVs-stimulated BV-2 microglial cells. BV-2 microglial cells were treated with NfEVs (50 µg/mL) with or without the pre-treatment of cerdulatinib. Phosphorylation levels of JAK1, JAK2, and STAT3 were analyzed via immunoblots using a specific antibody for each protein. Fold change (mean ± SD) means relative density change compared to negative control without treatment with NfEVs and inhibitor. (**d**) Crosstalk between MAPK and JAK-STAT pathway in NfEVs-treated BV-2 microglial cells. The cells were pre-treated with MAPK or JAK-STAT inhibitors, followed by activation with NfEVs. Phosphorylation levels of JNK, p38, ERK, and STAT3 were analyzed via immunoblots using a specific antibody for each protein. The β-actin was used as an internal control. Fold change (mean ± SD) means relative density change compared to negative control without treatment with NfEVs and inhibitor. *** *p* < 0.0001, ** *p* < 0.001, and * *p* < 0.01.

The mRNA and protein expression patterns of TNF-α, IL-6, and MIP-2 were analyzed to examine whether the JAK-STAT pathway could regulate proinflammatory responses in BV-2 microglial cells upon treatment with NfEVs. Pre-treatment with cerdulatinib (JAK-STAT inhibitor) inhibited the mRNA expressions of TNF-α, IL-6, and MIP-2 in a dose-dependent manner (Figure 8a). The inhibitor also gradually decreased their production at the protein level (Figure 8b). The phosphorylation status of JAK1, JAK2, and STAT3 was also analyzed. Immunoblot analysis showed that the phosphorylation levels of JAK1 and STAT3 increased in a time-dependent manner, whereas JAK2 phosphorylation was not detected (Figure 8c). The phosphorylation levels of JAK1 and STAT3 were significantly inhibited by pre-treatment with cerdulatinib, suggesting that JAK1-STAT3 is also involved in NfEVs-induced proinflammatory immune responses in BV-2 microglial cells (Figure 8c). To analyze the crosstalk between the MAPK and JAK-STAT signaling pathways in NfEVs-induced proinflammatory immune responses in BV-2 microglial cells, an additional immunoblot analysis was performed using the cells treated with each inhibitor. Each inhibitor blocked the phosphorylation of its corresponding target protein without affecting other proteins. Interestingly, ERK phosphorylation was suppressed by both cerdulatinib and U0126, suggesting that JAK-STAT activation may be associated with subsequent ERK pathway activation (Figure 8d).

## 3. Discussion

Potential pathological functions of excretory and secretory products (ESP) of *N. fowleri* as immune modulators and pathogenic factors have been reported previously [17,18]. However, studies with individual molecules in the ESP are necessary to recognize the critical molecules involving the pathogenesis of the amoeba and to understand their specific pathological or biological functions. The ESP of *N. fowleri* is likely to consist of diverse molecules such as proteins, lipids, and EVs. The potential roles of several molecules, including cathepsin Bs, have been investigated [18,20], but the role of EVs released from the amoeba has yet to be understood.

Parasitic protozoans secrete EVs that serve various biological functions essential for parasite survival and pathogenesis, as well as parasite–host interactions [4,5,6,7,8,9,10]. The cargo inside the protozoan EVs is transferred to host recipient cells through several mechanisms, such as receptor-mediated, fluid-phase endocytosis or direct fusion, resulting in modulation of the biological functions of the host cells, even death [11,21,22,23]. Because of these essential roles of EVs of protozoan parasites, there have recently been extensive studies aiming to understand the nature of protozoan EVs as pathogenic factors. In this study, we investigated the functional properties of NfEVs and found evidence that NfEVs can interact with host cells via internalization into host cells. Protozoan EVs are bilayered membrane structures with size variations that depend on parasite species, origin, cultivation conditions, and purification methods [8,21,24]. NfEVs also showed consistent broad size variations ranging from 22.4 to 955 nm, with a median size of approximately 186.3 nm. Recently, biophysical and biochemical properties of EVs isolated from a clinical isolate of *N. fowleri* were reported [25]. The sizes of NfEVs purified in this study were similar to EVs from a clinical isolate. Proteomic profile analysis of the EVs from clinical isolates of *N. fowleri* identified at least 184 proteins as part of the cargo [25]. We also tried proteome analysis of NfEVs and found that the vesicles contain diverse *N. fowleri*-derived proteins associated with biological processes, metabolic functions, and cellular components. However only small numbers of proteins could be clearly identified due to the poor resolution and low accuracy of MALDI-TOF, as well as limited information on *N. fowleri* proteins (Supplement File S3: Table S1). Some of them such as C2 domain-containing protein, peptidase proteins, and RGS domain-containing protein were matched with the previous study [25]. These evidences indicating the presence of diverse biological molecules in the NfEVs and internalization of the vesicles into C6 glial cells and BV-2 microglial cells suggest that the cargo of the vesicles was delivered into the cells. The internalization of NfEVs did not affect the viability of the recipient cells, implying that the contents released from the vesicles might modulate host cell functions without inducing direct cell death in the recipient cells.

To understand the biological functions of NfEVs, we analyzed the immune response of BV-2 microglial cells stimulated with NfEVs. Microglia are macrophage-like cells that are largely found in the brain and CNS and serve as part of the front line in immune defense by removing pathogens and damaged neuronal cells [26]. Microglia have also been reported to have important immune functions in *N. fowleri* [17,19,20,27,28]. NfEVs induced the production of various proinflammatory cytokines and chemokines in BV-2 microglial cells, coupled with the increased expressions of TLR-2, TLR-4, and MyD88. TLRs serve as pattern-recognition receptors and are important determinants of inflammatory responses, as well as specific downstream intracellular signaling cascades [29]. Studies have previously addressed the essential roles played by TLRs in host immune modulations associated with protozoan EVs [30,31,32,33]. Previous studies have also demonstrated the functional significance of the TLR pathway in immune regulation in host cells exposed to *N. fowleri* trophozoites or antigens [20,34]. The up-regulations of TLR-2, TLR-4, and MyD88 expression in NfEVs-stimulated BV-2 microglial cells suggest that the proinflammatory responses in these cells are mediated by MyD88-dependent TLR-2/TLR-4. In addition to the major proinflammatory cytokines, including TNF-α, IL-1α, IL-1β, IL-6, and IFN-γ, the expression of MIP-1α and MIP-2 was also profoundly increased, suggesting that the activation of BV-2 microglial cells by NfEVs could further promote the recruitment of myelomonocytic cells and/or polymorphonuclear neutrophils [35,36], which may exacerbate deleterious inflammation in *N. fowleri*-infected foci. Recently, a study reported the NfEVs-induced inflammatory response in THP-1 macrophages. NfEVs induced TNF-α and IL-8 expression in NfEVs-stimulated THP-1 cells but not in IL-1α, IL-6, and IL-10 [37]. This inconsistency could be attributed to the different experimental conditions and cell types used.

The enhanced phosphorylation of JNK, p38, and ERK and suppressed expression of TNF-α, IL-6, and MIP-2 at both the mRNA and protein levels by specific inhibitors of JNK, p38, and ERK suggest that MAPK is associated with NfEVs-induced proinflammatory immune responses in BV-2 microglial cells. The expression of proinflammatory cytokines was also downregulated by NF-κB and AP-1 inhibitors, implying that NfEVs evoke proinflammatory immune responses in BV-2 microglial cells via NF-κB and AP-1 dependent MAPK pathway. The enhanced phosphorylation and nuclear translocation of p65 supports the importance of NF-κB in this process. The finding that the phosphorylation status of JAK1 and STAT3 was markedly enhanced by NfEVs, and that the production of TNF-α, IL-6, and MIP-2 was downregulated by cerdulatinib, a JAK-STAT inhibitor, indicated that the JAK-STAT pathway is also involved in immune activation in NfEVs-stimulated BV-2 microglial cells. Interestingly, the JAK-STAT inhibitor led to concomitant reductions in the phosphorylation levels of ERK and STAT3, suggesting interdependent regulation of the ERK and JAK-STAT pathways. However, p38 and JNK activation was not linked to the JAK-STAT pathway. Considering that the ERK inhibitor did not inhibit STAT3 activation, it is likely that the JAK-STAT pathway acts upstream of ERK. Similar crosstalk between the ERK and JAK-STAT pathways has been found in ganglioside-stimulated BV-2 microglial cells [38]. Further studies should aim to better understand the molecular mechanisms underlying the crosstalk between JAK-STAT and ERK in NfEVs-stimulated BV-2 microglial cells.

The finding that NfEVs contribute to the pathogenesis of *N. fowleri* by inducing a proinflammatory immune response in microglial cells has significant implications for developing therapeutic interventions. Considering the significant impact of inflammation in exacerbating brain tissue damage in PAM, directing attention toward NfEVs may provide a promising avenue for developing novel therapeutic strategies. Designing therapeutics with the selective ability to inhibit or regulate the interaction between these molecules and microglia holds promise in reducing the excessive immune response and minimizing host brain damage.

## 4. Materials and Methods

### 4.1. Cultivation of Naegleria fowleri

*Naegleria fowleri* (Carter NF69 strain, ATCC 30215) was axenically cultured in Nelson’s media containing 5% heat-activated fetal bovine serum (FBS; Gibco, Grand Island, NY, USA) and 1% penicillin/streptomycin (P/S; Gibco, Grand Island, NY, USA) at 37 °C [39]. The trophozoites under the early logarithmic growth phase were harvested and used in all experiments.

### 4.2. Cultivation of BV-2 Microglial Cells and C6 Glial Cells

BV-2 mouse microglial cells and C6 rat glial cells (ATCC CCL-107) were maintained in Dulbecco’s Modified Eagle’s Medium (DMEM; Welgene, Daegu, Republic of Korea) supplement with 10% FBS (Gibco, Grand Island, NY, USA) and 1% P/S (Gibco, Grand Island, NY, USA). The cells were incubated at 37 °C in a humidified atmosphere containing 5% CO_2_, sub-cultured, and used in the study.

### 4.3. Isolation of NfEVs

*N. fowleri* trophozoites were cultured in Nelson’s medium until they reached 70% confluency. After removing the medium, the amoebae were washed three times with phosphate buffered saline (PBS, pH 7.4), resuspended in FBS-depleted Nelson’s medium, transferred to 175 cm^2^ cell culture flask (SPL Life Sciences, Gyeonggi, Republic of Korea), and incubated at 37 °C for 72 h (80% confluency). The supernatant (approximate 2 L) was harvested, and the floated cells and large debris were removed via centrifugation at 2500 rpm at 4 °C for 10 min. The collected supernatant was further filtered through a 0.22 µm syringe filter (Hyundai Micro, Seoul, Republic of Korea) to remove large residual particles. The cell-free media were then transferred to Centricon Plus-70 centrifugal filter device (100 kDa, Merck Millipore, Burlington, MA, USA) and centrifuged at 3000 rpm until the sample was concentrated (the final volume of 1 mL). In order to purify NfEVs, the 500 µL of the concentrated sample was applied to an qEV original 35 nm size exclusion column (Izon, Christchurch, New Zealand) and eluted following the manufacturer’s instruction. Eluted fractions (500 µL per each fraction) were collected, and the quality of all fractions was analyzed using 12% sodium dodecyl sulfate-polyacrylamide gel electrophoresis (SDS–PAGE) followed by silver staining (Thermo Fisher Scientific, Waltham, MA, USA). Protein concentration in each fraction was measured using Micro BCA assay (Thermo Fisher Scientific, Waltham, MA, USA). Based on the SDS–PAGE gel image and guidelines of the manufacturer, fractions harboring EVs (fractions 8 and 9) and secreted proteins (fractions 11 to 20) were confirmed. The fractions with high concentrations of NfEVs (fractions 8 and 9) were pooled and used for further study.

### 4.4. Dynamic Light Scattering (DLS) Analysis

Dynamic light scattering (DLS) analysis was performed to determine the size distributions of NfEVs. NfEVs size distribution profile was measured using a particle size and zeta potential analyzer (Zetasizer Nano ZS; Malvern Panalytical, Malvern, UK) at the Gyeongsang National University Center for Research Facilities (Jinju, Republic of Korea) under the set to detect populations ranging from 0.5 to 1000 nm. The size distribution of NfEVs was calculated using the intensity-weighted distribution obtained from the non-negative least squares (NNLS) algorithms. The measurements were conducted in duplicates at 25 °C for three independent replications. The median size and standard deviation (SD) of NfEVs were calculated from the three individual assays.

### 4.5. Transmission Electron Microscopy (TEM) Analysis

The purified NfEVs were washed three times with PBS and pelleted via centrifugation at 200× *g* for 5 min. The NfEVs were fixed 1:1 with 0.2% paraformaldehyde for 24 h. The NfEVs were pipetted onto 200-mesh copper grids (Agar Scientific, Stansted, UK) with carbon-coated formvar film and incubated for 5 min. The grid was placed on 2% uranyl acetate for 7 min. Morphological structures of NfEVs were acquired using a Talos L120C Cryo Bio 120 kV Transmission Electron Microscope (Thermo Fisher Scientific, Waltham, MA, USA) at the Gyeongsang National University Center for Research Facilities (Jinju, Republic of Korea).

### 4.6. Internalization Assay of NfEVs to BV-2 Microglial Cells and C6 Glial Cells

The purified NfEVs (100 µg/mL) were stained using 2 mM 1,1′-Dioctadecyl-3,3,3′,3′-tetramethylindodicarbocyanine-5,5′-disulfonic acid (DilC_18_(5)-DS; Invitrogen, Waltham, MA, USA) in PBS at 4 °C for 1 h and collected via centrifugation at 15,000 rpm at 4 °C for 50 min. The EVs pellets were then washed three times with PBS and resuspended into DMEM (Welgene, Daegu, Republic of Korea) medium. BV-2 microglial cells and C6 glial cells were seeded on a 24-well plate covered with sterile glass coverslips (10^5^ cells/well) and incubated at 37 °C, respectively. When the cells reached 80% confluency, NfEVs (20 µg/mL) were applied to the cells for 30 min, 1 h, and 2 h. At the indicated time points, the cells were washed with PBS twice to remove residual NfEVs, fixed with 3.7% formaldehyde for 40 min, and blocked with 1% bovine serum albumin (BSA; Sigma, St. Louis, MO, USA) in PBS at room temperature (RT) for 30 min. The fixed cells were labeled with 1 µg/mL of cholera toxin subunit B (CtxB; Invitrogen, Carlsbad, CA, USA) at 4 °C for 1 h. After two washes, the cells were labeled with 10 µg/mL of 4′,6-diamidino-2-phenylindole (DAPI; Thermo Fisher Scientific, Waltham, MA, USA) at RT for 30 min. The coverslips were rinsed with PBS three times, mounted with anti-fade fluorescence mounting medium (Abcam, Cambridge, UK), and observed using a EVOS™ M5000 Imaging System (Thermo Fisher Scientific, Waltham, MA, USA). The cells treated with DilC_18_(5)-PBS were used as negative controls.

### 4.7. Cell Viability Assay

To determine the potential cytotoxicity of NfEVs to mammalian cells, BV-2 microglial cells, C6 glial cells, and Chinese hamster ovary (CHO-K1; ATCC CCL-61) cells were seeded on 96-well microplates (2 × 10^4^ cells/well) and cultured at 37 °C overnight. Serially diluted NfEVs (10, 20, 50, or 100 µg/mL) were applied to the cells, respectively, and incubated at 37 °C for 48 h. The morphology of the cells was analyzed using a EVOS^TM^ XL Core Imaging System (Thermo Fisher Scientific, Waltham, MA, USA). Cell viability assay was performed using CellTiter-Blue Cell viability assay (Promega, Madison, WI, USA) according to the manufacturer’s protocols. The plate was read at a wavelength of 600 nm and a reference wavelength of 630 nm using a Multiskan FC microplate reader (Thermo Fisher Scientific, Waltham, MA, USA).

### 4.8. Analysis of Immune Responses in BV-2 Microglial Cells upon Treatment of NfEVs

BV-2 microglial cells were seeded in a 12-well plate (Thermo Fischer Scientific, Waltham, MA, USA) at a density of 10^5^ cells/well and cultured to about 70% confluence. After changing the media to fresh FBS-free DMEM media, NfEVs (50 μg/mL in FBS-free media) were added to the cells and incubated for 6 h and 9 h, respectively. The supernatants were harvested at the indicated time points and profiles of cytokines and chemokines induced by NfEVs were screened using Proteome Profiler Mouse Cytokine Array Panel A (R&D systems, Minneapolis, MN, USA). As negative controls, supernatant from the BV-2 microglial cells that were not stimulated with NfEVs were analyzed. The density of each spot was analyzed using ImageJ software ver. 1.52 [40]. The expression profiles of cytokines and chemokines were further analyzed via a quantitative reverse transcription polymerase chain reaction (RT-qPCR). Total RNA was isolated from the cells using RNAiso Plus (Takara, Otsu, Japan) following the manufacturer’s protocols. Purified total RNA was digested with RNase-free DNase (Takara, Otsu, Japan) to remove any contaminated DNA. The RNA concentration of each sample was measured via spectrophotometry (DeNovix DS-11; Wilmington, DE, USA), equalized, and used for cDNA synthesis using RNA to cDNA EcoDry Premix (Clontech, Mountain View, CA, USA). RT-qPCR was performed using primers specific for mouse toll-like receptor-2 (TLR-2), TLR-4, myeloid differentiation primary response 88 (MyD88), tumor necrosis factor-α (TNF-α), interleukin-1α (IL-1α), IL-1β, IL-6, IL-10, IL-17A, macrophage inflammatory protein-1α (MIP-1α), MIP-2, interferon-γ (IFN-γ), and glyceraldehyde-3-phosphate dehydrogenase (GAPDH) (Supplement File S4: Table S2). BV-2 microglial cells that were stimulated with lipopolysaccharides (LPS; 1 µg/mL, Sigma, St. Louis, MO, USA) and unstimualted cells were used as positive and negative controls, respectively. The protein levels of TNF-α, IL-6, and MIP-2 in the supernatant of the BV-2 microglial cells were measured via enzyme-linked immunosorbent assay (ELISA) using the Mouse Quantikine TNF-α ELISA kit (R&D Systems, Minneapolis, MN, USA), Mouse Quantikine IL-6 ELISA kit (R&D Systems, Minneapolis, MN, USA), and Mouse Quantikine MIP-2 ELISA kit (R&D Systems, Minneapolis, MN, USA) according to manufacturer’s protocols.

### 4.9. Analyses of Mitogen-Activated Protein Kinases (MAPKs), Nuclear Factor κB (NF-κB), and Janus Kinase-Signal Transducer and Activator of Transcription (JAK-STAT) Signaling Pathways

BV-2 microglial cells (10^5^ cells/well) were seeded in a 12-well plate and cultured as described above. To investigate signaling pathways involved in NfEVs-induced immune response in BV-2 microglial cells, the cells were pre-treated with different concentrations (1 µM or 10 µM) of each inhibitor for p38 (SB239063; Calbiochem, San Diego, CA, USA), c-Jun N-terminal kinase (JNK) (SP600125; Calbiochem, San Diego, CA, USA), extracellular signal-regulated protein kinase (ERK) (U0126; Calbiochem, San Diego, CA, USA), NF-κB (MG132; Calbiochem, San Diego, CA, USA), AP-1 (SR11302; Calbiochem, San Diego, CA, USA), or JAK-STAT (Cerdulatinib; Selleckchem, Houston, TX, USA) for 3 h, respectively. NfEVs (50 µg/mL) were applied to the cells and the cells were further incubated for 6 h and harvested. Expression levels of IL-1α, IL-1β, IL-6, MIP-1α, MIP-2, IFN-γ, and GAPDH were quantified using RT-qPCR as described above. Quantitative ELISA for TNF-α, IL-6, and MIP-2 was also performed using the same method described above. Phosphorylation statuses of proteins associated with MAPK, NF-κB, and JAK-STAT signaling pathways were analyzed via immunoblots. NfEVs (50 μg/mL) were added to the BV-2 microglial cells pre-treated with each inhibitor and the cells were cultured for 6 h and 9 h, respectively. The cells were harvested and lysed with RIPA buffer (Thermo Fisher Scientific, Waltham, MA, USA) [20]. Alternatively, cytosolic and nuclear proteins were isolated by using the ExKine Nuclear Protein Extraction Kit (Abbkine Inc., Wuhan, China). The total protein lysate, cytosolic protein, and nuclear protein were quantified by using a BCA protein assay kit (Thermo Fischer Scientific, Waltham, MA, USA). Protein samples (20 µg each) were analyzed via 10% sodium dodecyl sulfate-polyacrylamide gel electrophoresis (SDS–PAGE). Proteins were transferred onto nitrocellulose membrane (0.45 µm; GE Healthcare Life Science, Chicago, IL, USA) and the membrane was blocked with 5% skimmed milk in TBS supplemented with 1% Tween 20 (TBST). The membrane was incubated with either anti-TLR-2, anti-TLR-4, anti-MyD88, anti-β-actin, anti-Lamin A/C, anti-JNK, anti-p38, anti-ERK, anti-phospho-JNK, anti-phospho-p38, anti-phospho-ERK, anti-p65, anti-phospho-p65, anti-JAK-1, anti-phospho-JAK-1, anti-JAK-2, anti-phospho-JAK-2, anti-STAT3, or anti-phospho-STAT3 monoclonal antibodies (Cell Signaling Technology, Danvers, MA, USA) by 1:1000 dilution in TBST supplemented with 5% BSA (Sigma, St. Louis, MO, USA) at 4 °C overnight. The membrane was washed with TBST three times and incubated with horseradish peroxidase (HRP)-conjugated anti-rabbit IgG (Sigma, St. Louis, MO, USA) or HRP-conjugated anti-mouse IgG (Sigma, St. Louis, MO, USA). The immuno-reactive bands were visualized using the SuperSignal Pico PLUS chemiluminescent substrate (Thermo Fisher Scientific, Waltham, MA, USA). For internal controls, β-actin and Lamin A/C were used. The fold change of phosphorylated proteins compared with negative control was quantified using ImageJ software version 1.52.

### 4.10. Statisitcal Analysis

All experiments were performed in triplicate. Data are given as mean ± standard deviation (SD) of three independent experiments. Experimental differences were evaluated for statistical significance via a one-way ANOVA with Dunnett’s post hoc test using Prism 7 software (GraphPad Software, San Diego, CA, USA). A probability value of *p* < 0.01 was considered statistically significant.

## 5. Conclusions

**Figure 9:**
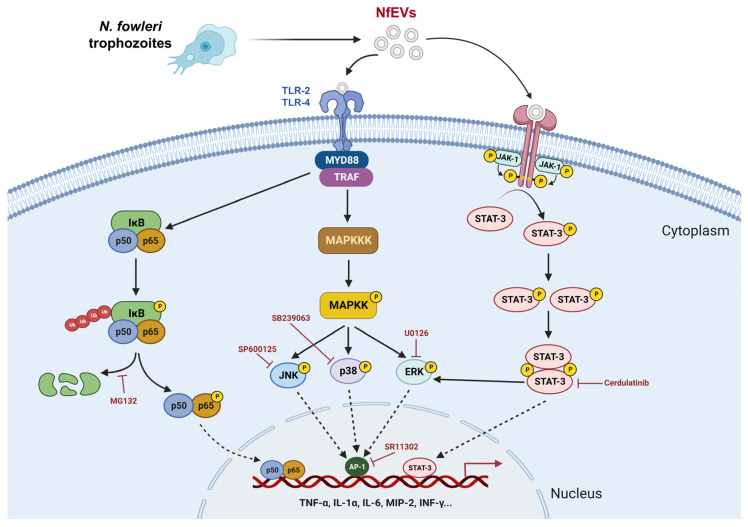
Summary of proinflammatory immune response in BV-2 microglial cells induced by NfEVs. NfEVs initially stimulate BV-2 microglial cells via the MyD88-dependent TLR-2/TLR-4 pathway. The NF-κB dependent MAPK pathways regulate the production of proinflammatory cytokines and chemokines in NfEVs-stimulated BV-2 microglial cells. Cerdulatinib, a JAK-STAT inhibitor, also downregulated phosphorylation of ERK, suggesting that the ERK is also affected by the JAK-STAT pathway. The image was created using BioRender (https://biorender.com/, accessed on 28 September 2022).

Evidence on the internalization of NfEVs into recipient host cells suggests that vesicles play potential roles in host–parasite interactions. NfEVs induce proinflammatory responses in BV-2 microglial cells, which are initiated by MyD88-dependent TLR-2/TLR-4. The enhanced expression of proinflammatory cytokines and chemokines by NfEVs is mediated via the NF-κB-dependent MAPK and JAK1-STAT3 signaling pathways. Crosstalk between ERK and JAK-STAT was also observed (Figure 9). It is not yet clear whether these proinflammatory immune responses in NfEVs-stimulated BV-2 microglial cells are evoked by the direct contact of NfEVs with the cells or by contents released from the vesicles into the cells, and this requires further elucidation. However, our findings suggest that NfEVs contribute to the pathogenesis of *N. fowleri* by activating proinflammatory immune responses in microglia, which could further induce subsequent inflammation cascades in other brain cells, such as astrocytes and glial cells, thereby resulting in deleterious inflammation in the *N. fowleri*-infected foci. The present study provides novel scientific insights into NfEVs and advances our knowledge of the contact-independent pathogenic mechanisms of *N. fowleri* in PAM. However, further studies are needed to better understand the nature of the components in NfEVs that induce proinflammatory immune responses in microglial cells, as well as the molecular basis of the immune responses induced by NfEVs.

## Data Availability

The data supporting the conclusions of this article are provided within the article and supplementary materials. The original datasets analyzed in the current study are available from the corresponding author upon request.

## Supplementary Materials

The following supporting information can be downloaded at: https://www.mdpi.com/article/10.3390/ijms241713623/s1.
