# Supplementary material for: Naegleria fowleri Extracellular Vesicles Induce Proinflammatory Immune Responses in BV-2 Microglial Cells
Source: Int J Mol Sci. 2023 Sep 3;24(17):13623. doi: 10.3390/ijms241713623 (PMC10487526; doi:10.3390/ijms241713623)

Figure S2. Effect of NfEVs on viability of mammalian cells. The cells were treated with different concentrations of NfEVs and morphological change (a) and cell viability (b) of the cells were examined.

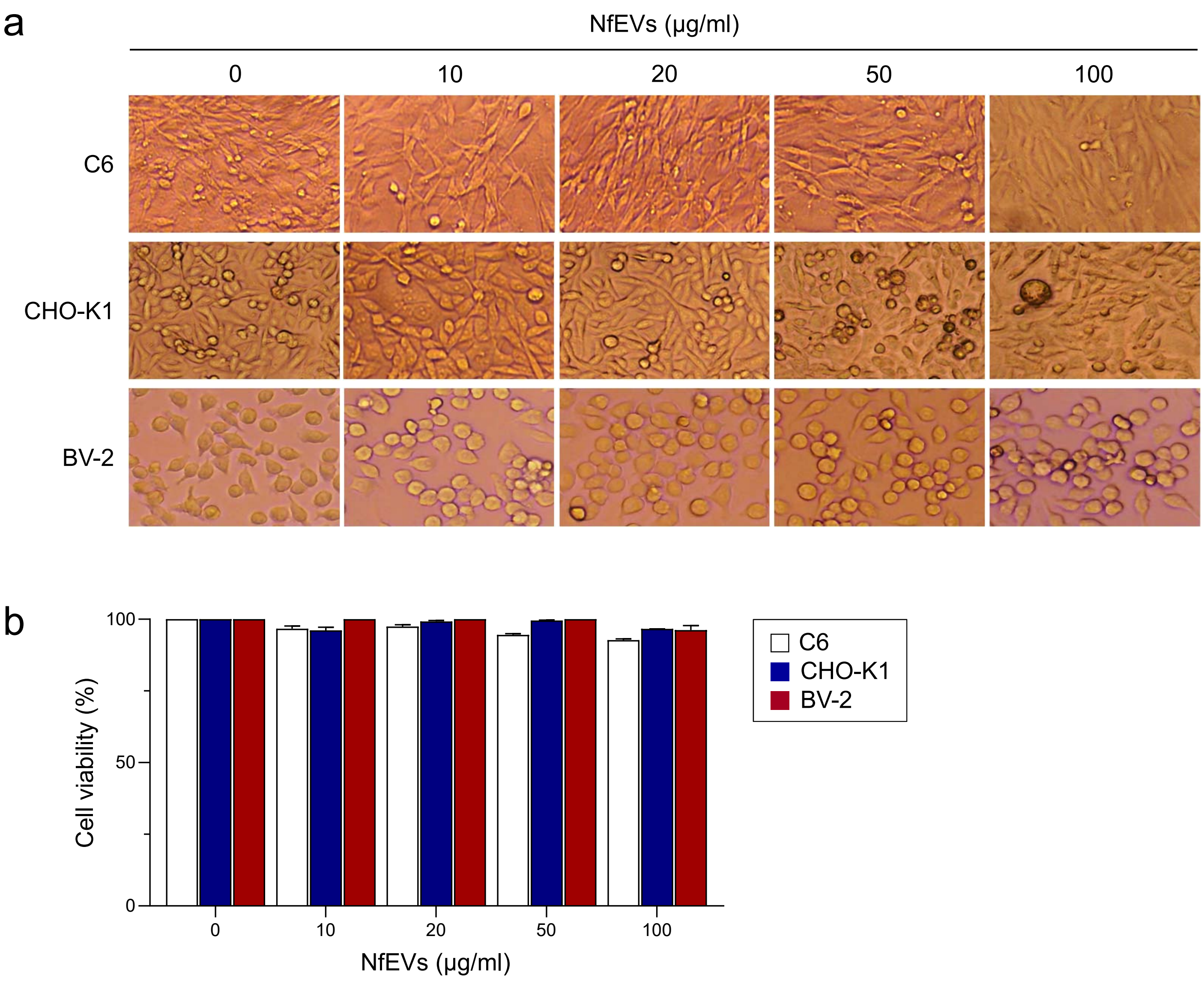

Supplement: Supplementary file 1 [file ijms-24-13623-s001.zip › Supplement File S2_Figure S2.pdf]
