# Supplementary material for: Naegleria fowleri Extracellular Vesicles Induce Proinflammatory Immune Responses in BV-2 Microglial Cells
Source: Int J Mol Sci. 2023 Sep 3;24(17):13623. doi: 10.3390/ijms241713623 (PMC10487526; doi:10.3390/ijms241713623)
Supplement: Supplementary file 1 [file ijms-24-13623-s001.zip › Supplement File S3_Table S1.pdf]

Table S1. List of identified NfEVs proteins in MALDI-TOF analysis

Entry	Entry name	Protein names	Gene names
A0A6A5CE41	A0A6A5CE41_NAEFO	AB hydrolase-1 domain-containing protein	FDP41_010634
A0A6A5BN48	A0A6A5BN48_NAEFO	ANAPC4_WD40 domain-containing protein	FDP41_004546
**A0A6A5C254***	**A0A6A5C254_NAEFO**	**C2 domain-containing protein**	**FDP41_012413**
A0A6A5BER1	A0A6A5BER1_NAEFO	Calponin-homology (CH) domain-containing protein	FDP41_009311
A0A6A5BZC1	A0A6A5BZC1_NAEFO	DNA helicase (EC 3.6.4.12)	FDP41_001064
A0A6A5C736	A0A6A5C736_NAEFO	Dymeclin	FDP41_010631
A0A6A5BUN3	A0A6A5BUN3_NAEFO	Epimerase domain-containing protein	FDP41_002636
A0A6A5BAZ8	A0A6A5BAZ8_NAEFO	ER membrane protein complex subunit 2	FDP41_006379
A0A6A5BX20	A0A6A5BX20_NAEFO	F-box domain-containing protein	FDP41_003197
A0A6A5BL46	A0A6A5BL46_NAEFO	F-box domain-containing protein	FDP41_008303
A0A6A5C3Y1	A0A6A5C3Y1_NAEFO	Gal_mutarotas_2 domain-containing protein	FDP41_012109
A0A2P1N6U3	A0A2P1N6U3_NAEFO	GOSR2	FDP41_012845
A0A6A5B9G4	A0A6A5B9G4_NAEFO	Guanylate cyclase domain-containing protein	FDP41_010035
A0A6A5CC11	A0A6A5CC11_NAEFO	LsmAD domain-containing protein	FDP41_008009
A0A6A5AY36	A0A6A5AY36_NAEFO	M16C_associated domain-containing protein	FDP41_010126
A0A6A5AW80	A0A6A5AW80_NAEFO	MHD domain-containing protein	FDP41_009501
A0A6A5BY13	A0A6A5BY13_NAEFO	N-acetyltransferase domain-containing protein	FDP41_001764
A0A6A5BUX9	A0A6A5BUX9_NAEFO	NIT domain-containing protein	FDP41_012805
A0A6A5C079	A0A6A5C079_NAEFO	Ntox11 domain-containing protein	FDP41_001127
**A0A6A5BDU6***	**A0A6A5BDU6_NAEFO**	**Pept_C1 domain-containing protein**	**FDP41_009444**
**A0A6A5BKY5***	**A0A6A5BKY5_NAEFO**	**Peptidase_S9 domain-containing protein**	**FDP41_002989**
A0A6A5BS95	A0A6A5BS95_NAEFO	PHD domain-containing protein	FDP41_001210
A0A6A5C8T6	A0A6A5C8T6_NAEFO	PPPDE domain-containing protein	FDP41_010913
A0A6A5BSI3	A0A6A5BSI3_NAEFO	Protein farnesyltransferase subunit beta (FTase-beta) (EC 2.5.1.58)	FDP41_003691
A0A6A5CDK6	A0A6A5CDK6_NAEFO	Protein kinase domain-containing protein	FDP41_010444
A0A6A5C789	A0A6A5C789_NAEFO	Protein kinase domain-containing protein	FDP41_000206
A0A6A5BR98	A0A6A5BR98_NAEFO	PUM-HD domain-containing protein	FDP41_003989
A0A6A5BPE1	A0A6A5BPE1_NAEFO	Ras-GEF domain-containing protein	FDP41_002027
**A0A6A5ATH1***	**A0A6A5ATH1_NAEFO**	**RGS domain-containing protein**	**FDP41_009946**

A0A6A5BSP9	A0A6A5BSP9_NAEFO	Ribosomal RNA-processing protein 4	FDP41_003887
A0A6A5C4V0	A0A6A5C4V0_NAEFO	RRM domain-containing protein	FDP41_012676
A0A6A5CE57	A0A6A5CE57_NAEFO	S1 motif domain-containing protein	FDP41_007683
A0A6A5CC84	A0A6A5CC84_NAEFO	SprT-like domain-containing protein	FDP41_010285
A0A6A5BZ85	A0A6A5BZ85_NAEFO	ThiF domain-containing protein	FDP41_001332
A0A6A5C9I9	A0A6A5C9I9_NAEFO	TLDc domain-containing protein	FDP41_011320
A0A6A5BI39	A0A6A5BI39_NAEFO	TRUD domain-containing protein	FDP41_004506
A0A6A5BQR9	A0A6A5BQR9_NAEFO	UDPGT domain-containing protein	FDP41_005122
A0A6A5CE39	A0A6A5CE39_NAEFO	UTP--glucose-1-phosphate uridylyltransferase (EC 2.7.7.9)	FDP41_000753
A0A6A5C6W5	A0A6A5C6W5_NAEFO	V-type proton ATPase proteolipid subunit	FDP41_010830
A0A6A5BXB4	A0A6A5BXB4_NAEFO	YL1_C domain-containing protein	FDP41_003307
A0A6A5C653	A0A6A5C653_NAEFO	Zn(2)-C6 fungal-type domain-containing protein	FDP41_012570

* Proteins matched with NfEVs proteome obtained in the previous study [25].
