# Supplementary material for: Naegleria fowleri Extracellular Vesicles Induce Proinflammatory Immune Responses in BV-2 Microglial Cells
Source: Int J Mol Sci. 2023 Sep 3;24(17):13623. doi: 10.3390/ijms241713623 (PMC10487526; doi:10.3390/ijms241713623)
Supplement: Supplementary file 1 [file ijms-24-13623-s001.zip › Supplement File S4_Table S2.pdf]

Table S2. Primer sequences of cytokines and chemokines

Gene	Primer sequences
TLR-2	Forward: 5′-TGCTTTCCTGCTGGAGATTT-3′<br>Reverse: 5′-TGTAACGCAACAGCTTCAGG-3′
TLR-4	Forward: 5′-GCATGGCTTACACCACCTCT-3′<br>Reverse: 5′-GTGCTGAAAATCCAGGTGCT-3′
MyD88	Forward: 5′-GCTGCTGGCCTTGTTAGACCGTGA-3′<br>Reverse: 5′-GACGTCACGGTCGGACACACACAAC-3′
TNF-α	Forward: 5′-CATCCTCTCAAAATTCGAGTGACA-3′<br>Reverse: 5′-TGGGAGTAGACAAGGTACAACCC-3′
IL-1α	Forward: 5′-ATGGCCAAAGTTCCTGACTT-3′<br>Reverse: 5′-TGGTCTTCTCCTTGAGCGCT-3′
IL-1β	Forward: 5′-TGCAGAGTTCCCCAACTGGTACAT-3′<br>Reverse: 5′-GTGCTGCCTAATGTCCCCTTGAAT-3′
IL-6	Forward: 5′-CCGGAGAGGAGACTTCACAG-3′<br>Reverse: 5′-GGAAATTGGGGTAGGAAGGA-3′
IL-10	Forward: 5′-TACCTGGTAGAAGTGATGCC-3′<br>Reverse: 5′-CATCATGTATGCTTCTATGC-3′
IL-17A	Forward: 5′-ATGAGTCCAGGGAGAGCTTC-3′<br>Reverse: 5′-TTAGGCTGCCTGGCGGACAATC-3′
MIP-1α	Forward: 5′-GACACTCTGCAACCAAGTCTTCTC-3′<br>Reverse: 5′-GGAACGTGTCCTGAAGTCTTTCAG-3′
MIP-2	Forward: 5′-AATGGCAACATCAGGTCGGCCATCACT-3′<br>Reverse: 5′-GCTGTGTGTCACAGAAGTCTCGAACTC-3′
IFN-γ	Forward: 5′-TGAACGCTACACACTGCATCTTGG-3′<br>Reverse: 5′-CGACTCCTTTTCCGCTTCCTGAG-3′
GAPDH	Forward: 5′-ACCAGAGTCCATGCCATCAC-3′<br>Reverse: 5′-CACCACCCTGTTGCTGTAGCC-3′
