# Supplementary figures and images for: Naegleria fowleri Extracellular Vesicles Induce Proinflammatory Immune Responses in BV-2 Microglial Cells
Source: Int J Mol Sci. 2023 Sep 3;24(17):13623. doi: 10.3390/ijms241713623 (PMC10487526; doi:10.3390/ijms241713623)

Figure S1: Fluorescence images of cells treated with DiIC<sub>18</sub>(5)-PBS.

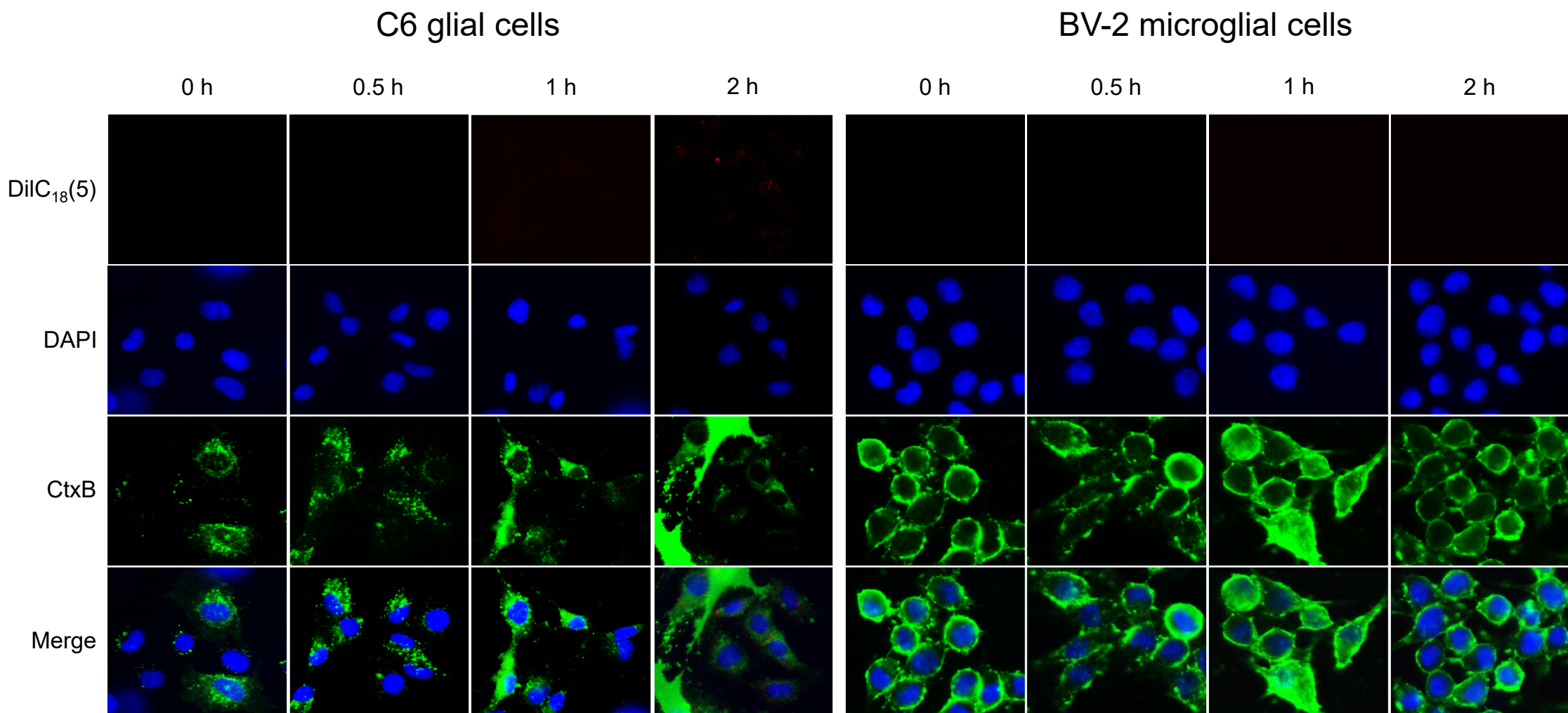

Supplement: Supplementary file 1 [file ijms-24-13623-s001.zip › Supplement File S1_Figure S1.pdf]
